# Hygiene practices: Are they protective factors for eczema symptoms?

**DOI:** 10.1002/iid3.217

**Published:** 2018-03-07

**Authors:** David Ferrandiz‐Mont, Nur Wahyuniati, Hsin‐Jen Chen, Mulyadi Mulyadi, Tjut Mariam Zanaria, Dar‐Der Ji

**Affiliations:** ^1^ International Health Program, Institute of Public Health, School of Medicine National Yang‐Ming University Taipei, Taiwan Republic of China; ^2^ Faculty of Medicine, Department of Parasitology Syiah Kuala University Aceh Indonesia; ^3^ Faculty of Medicine, Department of Pulmonology and Respiratory Medicine Syiah Kuala University Aceh Indonesia; ^4^ Department of Tropical Medicine, School of Medicine National Yang‐Ming University Taipei, Taiwan Republic of China

**Keywords:** Atopic dermatitis, eczema, epidermal barrier dysfunction, hygiene hypothesis, partial ablution

## Abstract

**Introduction:**

Exact etiology and proper treatment of eczema are still unknown. The hygiene hypothesis and epidermal barrier dysfunction hypothesis attempted to give some plausible explanations for these issues but they still remain unclear. The identification of factors, including hygiene practices, related to eczema symptoms (ES) could shed some light on these matters. Therefore, this study aimed to determine risk factors related to ES and the ES prevalence in two disparate areas in terms of urbanization in Aceh, Indonesia.

**Methods:**

A cross‐sectional study with convenience sampling was conducted among schoolchildren living in urban and rural Aceh. Data on ES, socio‐demographic characteristics, environmental factors, partial ablution and other hygiene related factors were collected by parental questionnaires. In addition, children's anthropometric measurements were also collected.

**Results:**

The prevalence of current ES in the study population was 21%. When stratifying by residency, the prevalence of ES in urban and rural area was 20.93% versus 21.05%. Partial ablution was independently associated with a reduced risk of ES (OR = 0.36; 95% CI 0.13–0.96). Important risk factors for ES were paternal history of allergic disease (OR = 4.09%; 95% CI 1.51–11.11) and belonging to the older group of schoolchildren (10–13 years old) (OR = 2.57; 95% CI 1.03–6.40).

**Conclusions:**

There were no significant differences in the prevalence of ES between urban and rural settings, and partial ablution had a protective effect on ES. These findings support the epidermal barrier dysfunction hypothesis as a possible pathway of eczema.

## Introduction

Eczema, also known as “atopic eczema” or “atopic dermatitis,” is a chronic inflammatory skin condition characterized by an itchy red rash 1 and it is particularly prevalent among children 2. Even though health professionals have been dealing with eczema for a long time, the exact etiology and proper treatment for it are still unclear 1. Eczema has a negative impact on the quality of life of the affected children and their families. Symptoms of eczema include soreness and itching, which can cause sleepless in over 60% of the patients. Tiredness and mood swing can appear as a result of sleep deprivation and can hamper the social life of these children, particularly in the school setting 3. Furthermore, this skin condition can also cause medical and economic burdens to the affected children's family 4.

The prevalence of child eczema is higher in affluent than non‐affluent countries 5. The prevalence in developed countries for instance Australia, New Zeeland, and United Kingdom is more than 20% whereas in developing countries such as Indonesia, Vietnam, and India is less than 10% 6. However, in the last decade, eczema prevalence is increasing particularly in developing countries 7. We can find some evidence of that in countries such as Indonesia (Bandung) where in 6 years the prevalence of children aged 13 and 14 years old having eczema increases from 0.4% to 7.5% and in Malaysia (Alor Setar) where the prevalence of children suffering from eczema increased from 8.7% to 26.1% in an interval of seven years 7. It has been reported that this increase of eczema in developing countries could be caused by the rapid urbanization 8. In line with this theory, eczema has been proven to be positively associated with urban and industrialized settings 9, 10, obesity 11, higher socioeconomic status 12, smaller family size 13, and being free of helminth infections 14, 15. This theory is consistent with the Hygiene Hypothesis, which states that limited exposure to microorganisms in industrialized countries due to improved sanitary conditions, would increase immune reactivity. This rise in immune reactivity promotes the development of allergic and autoimmune diseases such as eczema 16.

Hygiene Hypothesis attempted to shed light on the etiology of eczema, however many questions remains still unanswered 17. Recent advances supported the idea that the development of eczema is due to the interaction between both environmental and genetic factors 18, which causes epidermal barrier dysfunction 19, 20. Mutations of filaggrin (FLG), an important structural protein in the stratum corneum of the epidermis, has been identify as an important predisposing factor in the development of eczema. FLG mutation has been strongly associated with eczema in Europe 21 and with eczema and other allergic disorders jointly 22. A plausible explanation for this association is that in presence of FLG‐null mutations, stratum corneum experiences a reduced capacity to retain water that affects the skin's elasticity and mechanical resistance compromising the skin barrier function and causing epidermal barrier dysfunction 23. This can lead to the formation of gaps in the stratum corneum, which increases the risk of penetration of allergens and irritants 21 and facilitates colonization of numerous microorganisms, such us *Staphylococcus aureus* 24. *Staphylococcus aureus* has been associated with the severity of eczema 25, 26 and the mechanisms underlying the exacerbation of skin inflammation has been described 27, 28.

Determining which hypothesis explains better the pathway of eczema would help to find a more suitable treatment for ES. According to the epidermal barrier dysfunction hypothesis, eczema treatments should focus on skin barrier repair, irritants and allergens avoidance, and proper use of topical products. Therefore, hygiene practices such as showering and bathing, which remove allergens and sweat and create less favorable conditions for *S. aureus* colonization 28, should be encouraged 20, 29, 30, 31, 32, 33. Use of conventional soap and detergents in these practices are not recommended since can make ES worse 19, 34. Contrarily, in concordance with the Hygiene Hypothesis, hygiene practices should be performed with caution to maintain high exposure to microorganisms. This study aimed to identify risk factors associated to ES with special focus on hygiene practices. We hypothesize that the study of the role of hygiene practices and other factors on ES can give a better understanding on the etiology and treatment of eczema, which is between the push and pull of the hygiene hypothesis and epidermal barrier dysfunction hypothesis.

## Methods

### Study setting and population

This cross‐sectional study was performed between July and September, 2015 in Banda Aceh and Aceh Besar, Indonesia.

**Figure 1:**
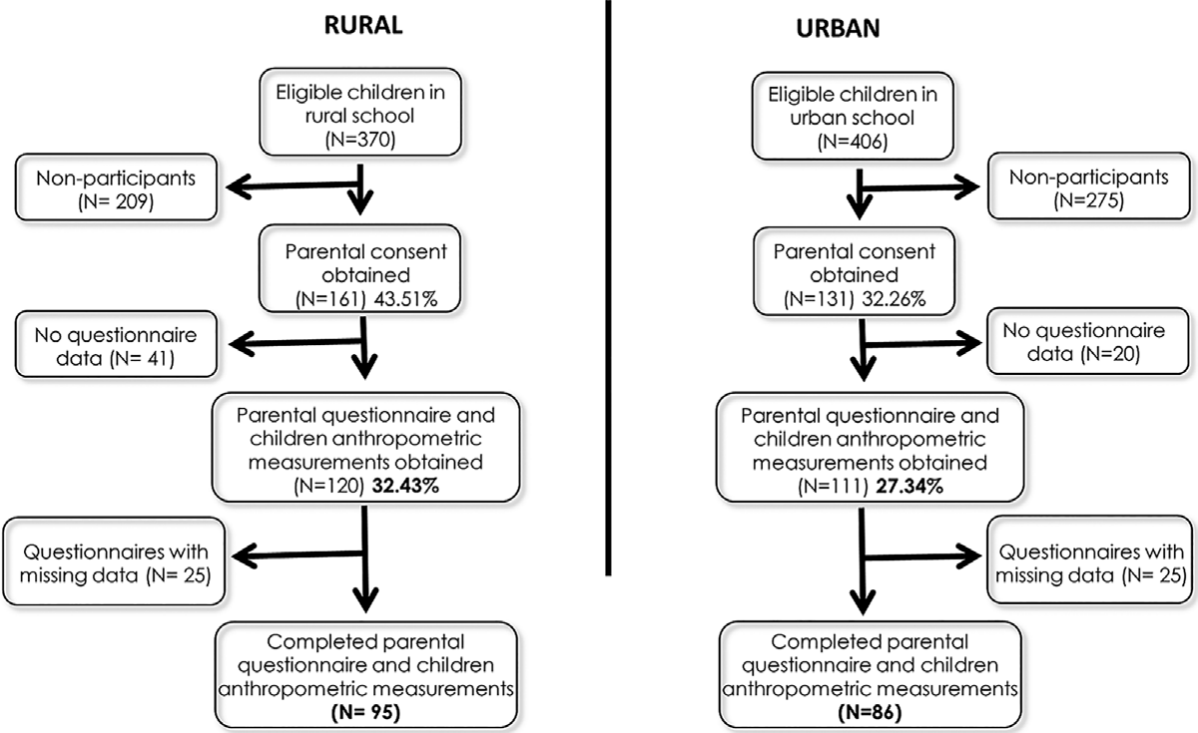
Flow diagram: recruitment of schoolchildren in urban and rural areas in Aceh Indonesia.

The study population was recruited from two elementary schools: one school was located in an urban area (Banda Aceh, the capital of Aceh province) and the other was in a rural area (Aceh Besar). School selection was done using convenience‐sampling method. A number of schools in urban and rural areas were considered, starting with the one nearest to the cooperative research center at Syiah Kuala University and taking into account the road conditions. The first school in urban and the first in rural that agreed to participate were included in this study. All the students who enrolled in the school at the time of study and their parents were invited to participate. The response rate was 27.34% (111 children) in the urban school and 32.43% (120 children) in the rural school (Fig. 1). After discarding incomplete questionnaires, the final number of children participating in the present study was 181.

### Data collection and outcome measurement

Data on eczema symptoms, socio‐demographic characteristics, behavioral factors, and environmental factors, including hygiene factors, were collected by parental questionnaires and data on children's nutritional parameters (height and weight) and personal information (age, gender, and grade) were collected by children's questionnaires. Specific questions from the International Study of Asthma and Allergy in Childhood (ISAAC) questionnaires 35 were selected and slightly modified to assess the eczema symptoms in our study setting. Parental and children's questionnaires were translated from English to Bahasa Indonesian and back to English to check for consistency. A pilot test was conducted among a small group of parents from the target population to check the validity of the questionnaires, which would later be modified based on the received feedback. Parental take‐home questionnaires were distributed to the schoolchildren taking part in the research and returned once they were filled out by parents or guardians.

The presence of current ES was determined by a positive response to an itchy flexural rash in the last 12 months, as described in previous studies 6, 7, 36. The question used to identify the cases was “In the past 12 months, has your child ever complained about itchy‐rash skin problem?”, it then had to be followed also by a positive response to the question “Has your child complained about itchy‐rash skin or dryness and scaly at the following places: elbows, behind the knees, around the ankles, under the buttocks, around the neck, ears or eyes?”

### Risk factor assessment

#### Hygiene practices

Partial ablution, also known as “wudhu,” is a religious ritual performed by the Muslim community, which involves cleaning the exposed parts of the body to the environment, such as face, nose, ears, hands and feet, and it only uses running clean water without soaps or chemicals. This religious ritual involves cleansing the body before prayers, which happen five times a day. Partial ablution performance variable was constructed based on the question: “Is your child able to perform partial ablution properly with or without your help?” The answers ranged from “never,” “sometimes,” and “always.” These answers were dichotomize into groups: “frequent partial ablution performance” (always) and “infrequent partial ablution performance” (never and sometimes).

Hand‐washing practices were compiled in a scale. Parents were asked the following questions regarding their children's hand‐washing habits:” Does your child wash his/her hands before eating?”, ”Does your child usually wash his/her hands with soap?” and “Does your child wash his/her hands after defecation?” The answers to these questions ranged from “Never,” “Sometimes,” and “Always.” They were given the score 0–2 and were compiled to create a scale (from 0 to 6 points). Finally, this scale was dichotomized into “infrequent hand‐washing” (from 0 to 5 points) and “frequent hand‐washing” (6 points).

#### Other factors

Height and weight measurements were performed at the school using a professional weighing scale (SMIC health scale, model ZT‐120). The body mass index (BMI) was calculated using BMI‐for‐age tables for boys and for girls from 5 to 19 years old (z‐scores) 37.

Social economic status (SES) was calculated based on the type of toilet (flush toilet or latrine/no toilet), water supply (treated/untreated) and the quantitative assessment of different assets according to the social reality of setting of the study (number of computers, refrigerators, vehicles, televisions, and mobiles phones). Factor analysis was performed with this information and then the SES was divided into three categories (tertiles) to define the range of high, middle and low socioeconomic status.

### Statistical analysis

Stata 13 (StataCorp LP, College Station, TX) was used for statistical analysis. Chi‐square tests were performed to compare differences between children who had experienced ES and children who had not. Univariate logistic model examines the crude association between the explanatory variable and the outcome, while multivariable logistic model examines the independent association between the explanatory variable and the outcome after all the other variables are adjusted for. *p* values <0.05 was considered statistically significant.

### Ethical consideration

Written consent forms were signed by the parents/guardians and the schoolchildren. Ethical Approval from Syiah Kuala University Aceh Indonesia ethical review board was obtained before the exercise.

## Results

### Characteristics of the study population

The study was based on a complete data of 181 schoolchildren in Aceh, Indonesia: 86 (47.51%) children from the urban area and 95 (52.49%) from the rural area. Among these children, the average age at enrollment was 8.70 (SD = 1.69), from which 55.24% were males; 120 children (66.30%) were between 6 to 9 years old and 61 children (33.70%) were between 10 and 13 years old.

### Prevalence of ES

The prevalence of ES in our study population was 21%. When stratified by residency (urban *vs*. rural), the prevalence of ES was slightly higher among rural children (21.05%) compared with urban children (20.93%), whereas when stratified by children age group (6–9 vs. 10–13), the prevalence were higher in the older group (26.23%) compared to the younger group (18.33%). However, these differences were not statistically significant.

### Risk factors of ES

The distribution of selected risk factors for ES are shown in Table 1. Children who had ES had significantly higher prevalence of paternal history of allergy, and were more likely to have a pet at home, compared to those who did not have ES (47.37% vs. 18.18% and 23.68% vs. 11.19%, respectively). In addition to this, at the time of the study, children who did not experience ES performed partial ablution more frequently than children who experienced ES (78.32% vs. 63.16%), but this difference did not reach the significance level.

**Table 1:** Characteristics of schoolchildren stratified by eczema symptoms status

Variables	No eczema symptoms *n* = 38 (20.99%)	Eczema symptoms *n* = 143 (79.01%)	X2 test: *p* value
Children characteristics			
Residency			
Urban area	68 (47.55)	18 (47.37)	0.984
Age			
6‐9 yrs	98 (68.53)	22 (57.89)	0.218
10‐13 yrs	45 (31.47)	16 (42.11)	
Mean (SD)	8.66 (1.68)	8.84 (1.74)	
Gender			
Male	81 (56.64)	19 (50.00)	0.464
Nutritional status			
Overweight	26 (18.18)	10 (26.32)	0.264
Parents/Guardian characteristics			
Education status			
Low education	84 (58.74)	24 (63.15)	0.928*
Middle education	16 (11.19)	4 (10.53)	
High education	43 (30.07)	10 (26.32)	
Socioeconomic Status			
Low	53 (37.06)	16 (42.10)	0.764
Middle	44 (30.77)	12 (31.58)	
High	46 (32.17)	10 (26.32)	
Hygiene factors			
Habits related to hygiene			
Hand‐washing performance			
Frequent	67 (46.85)	15 (39.47)	0.417
Partial ablution performance (PA)			
Frequent	112 (78.32)	24 (63.16)	0.055
Household characteristics related to hygiene			
Crowding			
(children ≤13 years old in the house)			
3 or more children	39 (27.27)	16 (42.11)	0.077
Pet at home			
Yes	16 (11.19)	9 (23.68)	0.047
Household water supply			
Treated water	64 (45.07)	17 (44.74)	0.971
Family allergic history			
Paternal allergic disease			
Probabily/Ever	26 (18.18)	18 (47.37)	>0.001
Maternal allergic disease			
Probabily/Ever	34 (23.78)	14 (36.84)	0.105
Others			
Smoker at home			
Yes	96 (67.13)	24 (63.16)	0.645
Breastfeeding			
>6 months	123 (86.01)	31 (81.58)	0.495

*Fisher exact test.

**Figure 2:**
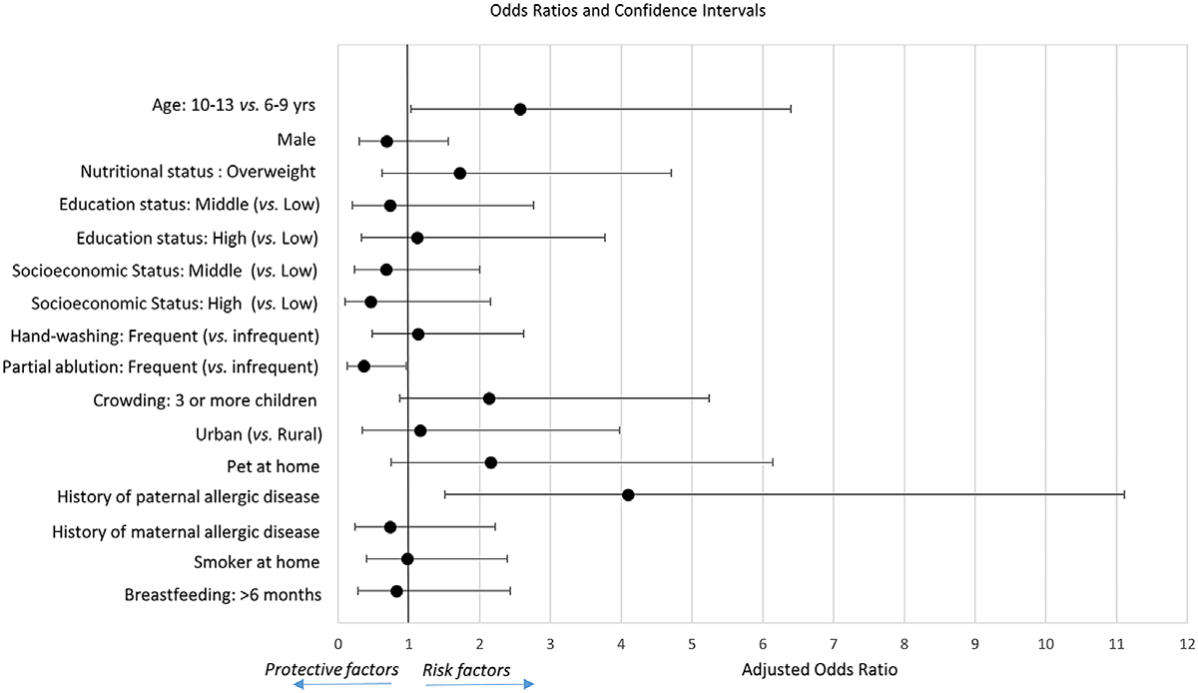
95% Confidence Interval plot: Protective and risk factors for eczema symptoms.

Univariate and multivariable analyses of factors associated with ES are shown in Table 2 and Figure 2. In univariate analysis, having paternal history of allergic disease was a risk factor for ES (OR = 4.05; 95% CI 1.88–8.71). When adjusting for potential confounders, frequent partial ablution performance was significantly associated with lower odds of ES (OR = 0.36; 95% CI 0.13–0.96). Paternal history of allergic disease remained associated with an increased risk of ES (OR = 4.09; 95% CI 1.51–11.11), and children aged between 10 and 13 years old became more likely to suffer from ES than those aged between 6 and 9 years (OR = 2.57; 95% CI 1.03–6.40).

**Table 2:** Univariate and multivariable analysis of factors associated with eczema symptoms

	Current eczema symptoms			
Variables	Single variable		Multivariable	
Children characteristics	OR	95% CI	OR	95% CI
Age				
10–13 yrs (vs. 6–9 yrs)	1.58	(0.76–3.30)	2.57	(1.03–6.40)
Gender				
Male	0.77	(0.37–1.57)	0.69	(0.30–1.56)
Nutritional status				
Overweight (vs. non‐overweight)	1.61	(0.69–3.71)	1.72	(0.62–4.71)
Parents/Guardian characteristics				
Education status				
Middle (vs. Low)	0.88	(0.27–2.86)	0.74	(0.20–2.76)
High (vs. Low)	0.81	(0.36–1.85)	1.12	(0.33–3.77)
Socioeconomic status				
Middle (vs. Low)	0.90	(0.39–2.11)	0.68	(0.23–2.00)
High (vs. Low)	0.72	(0.30–1.74)	0.46	(0.10–2.15)
Hygiene factors				
Habits related to hygiene				
Hand‐washing performance				
Frequent (vs. infrequent)	0.74	(0.36–1.53)	1.13	(0.48–2.62)
Partial ablution performance (PA)				
Frequent (vs. infrequent)	0.47	(0.22–1.02)	0.36	(0.13–0.96)
Household characteristics related to hygiene				
Crowding (children ≤13 years old in the house)				
3 or more children	1.94	(0.92–4.07)	2.13	(0.87–5.24)
Residency				
Urban (vs Rural)	0.99	(0.48–2.03)	1.16	(0.34–3.98)
Pet at home				
Yes	2.46	(0.99–6.12)	2.16	(0.75–6.14)
Family allergic history				
Paternal allergic disease				
Probably/ever	4.05	(1.88–8.71)	4.09	(1.51–11.11)
Maternal allergic disease				
Probably/ever	1.87	(0.87–4.01)	0.74	(0.24–2.22)
Others				
Smoker at home				
Yes	0.84	(0.40–1.77)	0.98	(0.40–2.39)
Breastfeeding				
>6 months	0.72	(0.28–1.85)	0.83	(0.28–2.43)

## Discussion

This study aimed to identify risk factors for ES with special focus on the study of hygiene factors such as handwashing and partial ablution. This study can give a better understanding of which hypothesis, either Hygiene Hypothesis or epidermal barrier dysfunction hypothesis, can contribute to a greater extend to explain the etiology of eczema. The findings of this study supported epidermal barrier dysfunction hypothesis over Hygiene Hypothesis in several aspects.

First, partial ablution was significantly associated with a reduced risk of ES, which supports the idea that partial ablution could be beneficial to protect the skin and decrease eczema symptoms. This finding suggests that frequent rinsing of the parts of body that are exposed to the environment, such as face, nose, ears, hands and feet, may remove allergens from the skin. Therefore, partial ablution may prevent the penetration of these agents through the skin and further inflammation 38. In addition, partial ablution could decrease sweat on the skin that causes itch and aggravates eczema symptoms 39. This could be particularly important in countries with tropical climates like Indonesia. Moreover, conventional soaps, detergents and other chemicals, which could be harmful for the epidermal barrier 19, 34, are not allowed during partial ablution performance and only running clean water is used. This could explain why hand‐washing habit that usually involves soap, did not reach the statistical significance as a protective factor for eczema symptoms. However, one should mention that use of soaps and detergents should not be discouraged but they should be applied with caution, particularly in the presence of epidermal barrier abnormality such as eczema 19.

Furthermore, there were no significant differences between urban and rural areas in terms of the prevalence of eczema symptoms. These findings are consistent with previous studies carried out in developed and developing countries 40, 41, 42 and they are controversial with the Hygiene Hypothesis, which would expect significant differences on eczema symptoms due to the distinct exposure to microorganisms between urban and rural areas 9, 10. Therefore, these results rather than being elucidated by the Hygiene Hypothesis, should find a plausible explanation on the complex interplay between both environmental and genetic factors in line with the epidermal barrier dysfunction hypothesis.

Finally, the genetic component of the epidermal barrier dysfunction hypothesis, maybe due to FLG‐null mutation, could support our finding that paternal history of allergic disease is a risk factor for ES. This finding is supported by several studies, which found that parental atopy is a risk factor for eczema 43, 44, 45; however, it differs from one study that did not find a significant relationship between parental FLG status and the manifestation of atopic dermatitis in the offspring 46.

This study also reveals that the older age group of children (10–13 years old) were more likely to suffer from ES than the younger age group (6–9 years old). This finding is not consistent with previous studies carried out in Asia 47, 48. One explanation for our findings is that even though the prevalence of eczema symptoms are showed to be higher among younger children in previous studies, the severity of these symptoms have been found to increase over time since eczema became exacerbated with increased age 47. Another possible explanation could lie in recent studies’ findings that higher prevalence of ES was found among children who had experienced a natural disaster 49, 50. The majority of older children in the present study might have experienced the 2004 tsunami in Aceh in a very early age, whereas younger children did not experience it. However, this should be further investigated.

Interestingly, the prevalence of ES in the present study was higher than in other studies in Indonesia using ISAAC questionnaire. In 2002 the prevalence of current eczema symptoms among 6–7 and 13–14 years old children in Indonesia was 3.7% and 3.1%, respectively, and the prevalence in the whole Asia‐Pacific region was 10.1% and 5.3%. In the present study the prevalence in the younger (6–9 years) and older group (10–13) of children was 18.33% and 26.23%, respectively. However, it should be noted that Indonesia comprises 34 provinces with very distinct location, climate, ethnicity, religion and culture, including Aceh Province. ISAAC questionnaires were only conducted in three major cities in Indonesia, located in Java and Bali Island, and which may not represent the whole country. Other countries such as Colombia (Barranquilla) or Nicaragua (Managua) reported prevalence, for 6–7 and 13–14 years old children, that are more in accordance with the present study (20.9% vs. 24.6% and 20.0% vs. 20.4%, respectively) 6, 7.

For all the aforementioned reasons, our findings support the epidermal barrier dysfunction hypothesis that encompasses genetic and environmental factors, as a possible pathway for eczema. However, the present study bears some limitations that should be acknowledged. This study could not assess important factors related to the Hygiene Hypothesis, such as the influence of parasitic infections since the burden of parasitic infections in our study population was very low (6.63%) due to the school‐based deworming campaigns at the target schools. Second, the use of questionnaires to assess allergy symptoms and risk factors could lead to information bias because it was reported by the parents. For example, they may be more prompt to report a frequent ablution performance for social desirability reasons. However, the over‐reporting ablution would not differ by allergy status group as all parents will report the same way the frequency of ablution. Third, the response rate of this study was low (around 30%), even though we sent reminders to all parents to respond and the teachers help ensure that most of the children were present the recruitment days. Moreover, in the urban school where the response was the lowest, there were no systematic differences in the sex and age distributions between respondents and non‐respondents. The fourth limitation was the capacity of generalizing the findings of this research. However, we recruited children from rural and urban areas to increase the heterogeneity of the convenience sample, attempting to make the results more representative to improve the sample's generalizability to the population of Aceh. The vast majority of the Acehnese population share a similar culture, deeply rooted in the Islamic values and religion; therefore, we may speculate that our findings may reflect schoolchildren in Aceh Province, in both rural and urban areas. Nevertheless, future surveys that take representative sampling are warranted. Finally, this study presents limitations related to its cross‐sectional design, which makes it difficult to infer causal‐relationships. Despite this, the present study has strengths. First, compared to the rest of Indonesia, Aceh province has the largest proportion of Muslims in the whole Indonesia, which can give new insights in the field of eczema, particularly regarding hygiene practices related to Islam such as partial ablution. Furthermore, we studied the effect of hygiene related exposures on eczema symptoms, which are not well understood in developing countries. In addition to this, the present study is the first study assessing the relationship between partial ablution and eczema, which can give interesting insights for future eczema treatments. Finally, this research could be an important starting point for future research in this field.

In conclusion, this study, conducted in the urban and rural areas of Aceh revealed that frequent hygiene practices that does not involve use of soaps and detergents, such as partial ablution, are protective for eczema symptoms. This association maybe explained from the epidermal barrier dysfunction hypothesis standpoint, which is based on the interaction between genetic and environmental factors. These findings could answer questions in existing literature, regarding treatments of eczema however, further studies, probability with a randomized trial design, should be done to confirm these findings.

## Conflict of Interest

The authors declare no conflict of interest.
